# The Effect of Quadriceps Muscle Length on Maximum Neuromuscular Electrical Stimulation Evoked Contraction, Muscle Architecture, and Tendon-Aponeurosis Stiffness

**DOI:** 10.3389/fphys.2021.633589

**Published:** 2021-03-29

**Authors:** Jonathan Galvão Tenório Cavalcante, Rita de Cassia Marqueti, Jeam Marcel Geremia, Ivo Vieira de Sousa Neto, Bruno Manfredini Baroni, Karin Gravare Silbernagel, Martim Bottaro, Nicolas Babault, João Luiz Quagliotti Durigan

**Affiliations:** ^1^Graduate Program in Rehabilitation Sciences, Faculdade de Ceilândia, Centro Metropolitano, Universidade de Brasília, Brasília, Brazil; ^2^Laboratório de Pesquisa do Exercício, Escola de Educação Física, Fisioterapia e Dança, Universidade Federal do Rio Grande do Sul, Porto Alegre, Brazil; ^3^Graduate Program in Sciences and Technology in Health, Faculdade de Ceilândia, Centro Metropolitano, Universidade de Brasília, Brasília, Brazil; ^4^Graduate Program in Rehabilitation Sciences, Universidade Federal de Ciências da Saúde de Porto Alegre, Porto Alegre, Brazil; ^5^Department of Physical Therapy, University of Delaware, Newark, DE, United States; ^6^Departamento de Educação Física, Faculdade de Educação Física, Universidade de Brasília, Brasília, Brazil; ^7^Unité Cognition, Action, et Plasticité Sensorimotrice, Faculté des Sciences du Sport, Université Bourgogne, Dijon, France

**Keywords:** exercise physiology, neuromuscular electrical stimulation, moment-angle relationship, muscle architecture, tendon-aponeurosis complex

## Abstract

Muscle-tendon unit length plays a crucial role in quadriceps femoris muscle (QF) physiological adaptation, but the influence of hip and knee angles during QF neuromuscular electrical stimulation (NMES) is poorly investigated. We investigated the effect of muscle length on maximum electrically induced contraction (MEIC) and current efficiency. We secondarily assessed the architecture of all QF constituents and their tendon-aponeurosis complex (TAC) displacement to calculate a stiffness index. This study was a randomized, repeated measure, blinded design with a sample of twenty healthy men aged 24.0 ± 4.6. The MEIC was assessed in four different positions: supine with knee flexion of 60° (SUP60); seated with knee flexion of 60° (SIT60); supine with knee flexion of 20° (SUP20), and seated with knee flexion of 20° (SIT20). The current efficiency (MEIC/maximum tolerated current amplitude) was calculated. Ultrasonography of the QF was performed at rest and during NMES to measure pennation angle (θ_*p*_) and fascicle length (*L*_*f*_), and the TAC stiffness index. MEIC and current efficiency were greater for SUP60 and SIT60 compared to SUP20 and SIT20. The vastus lateralis and medialis showed lower θ_*p*_ and higher *L*_*f*_ at SUP60 and SIT60, while for the rectus femoris, in SUP60 there were lower θ_*p*_ and higher *L*_*f*_ than in all positions. The vastus intermedius had a similar pattern to the other vastii, except for lack of difference in θ_*p*_ between SIT60 compared to SUP20 and SIT20. The TAC stiffness index was greater for SUP60. We concluded that NMES generate greater torque and current efficiency at 60° of knee flexion, compared to 20°. For these knee angles, lengthening the QF at the hip did not promote significant change. Each QF constituent demonstrated muscle physiology patterns according to hip and/or knee angles, even though a greater *L*_*f*_ and lower θ_*p*_ were predominant in SUP60 and SIT60. QF TAC index stiffened in more elongated positions, which probably contributed to enhanced force transmission and slightly higher torque in SUP60. Our findings may help exercise physiologist better understand the impact of hip and knee angles on designing more rational NMES stimulation strategies.

**Clinical Trial Registration:**

www.ClinicalTrials.gov, identifier NCT03822221.

## Introduction

Neuromuscular electrical stimulation (NMES) has been applied to increase, or attenuate loss, in muscle strength and size (Thomas and Stevens-Lapsley, 2012; Baroni et al., 2013a; Vaz et al., 2013). Adjusting joint angles may be a key strategy to increase training load with the lowest possible current amplitude (Herzig et al., 2015), referred to as current efficiency (torque/current amplitude), which is desirable to reduce the perceived discomfort during NMES (Lieber and Kelly, 1991; Medeiros et al., 2017). Thus, exercise physiologists and clinicians should choose joint angles that allow the generation of the highest evoked torques induced by NMES (Fahey et al., 1985; Herzig et al., 2015; Nussbaum et al., 2017), according to the force-length and moment-angle relationships (Gordon et al., 1966; Herzog et al., 1990).

The length of the knee extensor quadriceps femoris muscle (QF), often targeted for NMES (Maffiuletti, 2010), depends on both hip and knee joint angles (Glenn and Samojla, 2002). Few studies have assessed the maximum electrically induced contraction (MEIC) as a function of hip or knee joint angles. For instance, greater MEIC was found at 60° of knee flexion (Bremner et al., 2015; Scott et al., 2019), demonstrating a similar pattern observed during maximum voluntary contraction (MVC) (Gerrits et al., 2005). The MEIC of knee extension was also greater when participants were supine compare to seated while maintaining the knee at 90° (Maffiuletti and Lepers, 2003; Bampouras et al., 2017), suggesting that stretching the biarticular rectus femoris (RF) enhances the torque output. Besides, other mono-articular constituents could be affected by hip angle through intermuscular connections with the RF (Grob et al., 2018). These assumptions, however, have not been assessed considering important aspects of the muscle-tendon unit physiology like the muscle architecture and the tendon-aponeurosis complex (TAC) stiffness.

Muscle architecture variables are a strong determinant of muscle function (Lieber and Friden, 2000; Aagaard et al., 2001). Fascicle length (*L*_*f*_) is associated with the number of sarcomeres in series, which determines fiber/muscle shortening velocity and excursion (Abe et al., 2000; Blazevich, 2006; Thom et al., 2007). Pennation angle (θ_*p*_) is related to the parallel number of sarcomeres within the fiber and determines the maximal capacity for force transmission efficiency (Fukunaga et al., 1997; Blazevich and Sharp, 2005). An increase in θ_*p*_ is expected with a concomitant reduction in *L*_*f*_ during isometric contraction, with the extent depending on contraction intensity and muscle resting length (Fukunaga et al., 1997; Reeves and Narici, 2003; Ando et al., 2016). Adaptations in *L*_*f*_ and θ_*p*_ are found after physical training (Reeves et al., 2009; Baroni et al., 2013b; Geremia et al., 2019) and rehabilitation (Vaz et al., 2013). Interestingly, different constituents in a muscle group may adapt differently according to functional demands and joint angle (Abe et al., 2000; Mulder et al., 2009; Brughelli et al., 2010; Alegre et al., 2014; Ando et al., 2016; Geremia et al., 2019). Also, the TAC stiffness indicates the elongation from the deep aponeurosis to the distal free tendon in response to transmission of muscle force to bones (Kubo et al., 2006; Burgess et al., 2008). Joint angles that remove the slack of the TAC (increasing its stiffness) and optimize muscle length for greater force production seem to be ideal to speed up adaptation (Fukutani et al., 2017; Massey et al., 2018). Thus, exploring the QF muscle-tendon unit during NMES may inform part of the mechanisms by which MEIC and current efficiency may differ according to hip and knee angles. This knowledge will help exercise physiologists and clinicians to better understand the effects of NMES on the neuromuscular system and will contribute to an evidence-based upon which to develop NMES strategies.

The primary aim of this study was to investigate the effect of quadriceps muscle length by manipulating hip (0° or 85°) and knee (60° or 20°) joint knee angles on MEIC and current efficiency by NMES. We secondarily investigated the muscle architecture (θ_*p*_ and *L*_*f*_) at rest and during NMES of the four QF constituents: RF, vastus lateralis (VL), vastus medialis (VM), and vastus intermedius (VI), and the QF TAC stiffness. We hypothesized that: (1) during NMES, greater knee extensor torque and current efficiency would be obtained with the hip at 0° and the knee at 60° of flexion; (2) at rest and during the plateau of the evoked contraction, θ_*p*_ would be lower and *L*_*f*_ would be greater when the knee is at 60°, although for RF, the hip at 0° would decrease the θ_*p*_ and increase the *L*_*f*_ even more; and (3) the TAC stiffness would be greater at more elongated positions compared to shortened positions, as predicted for other muscle groups (Fukutani et al., 2017).

## Materials and Methods

### Trial Design

This was a randomized, repeated measure, single blinded study. We recruited twenty men of age: 24.0 ± 4.6 years, body mass: 77.0 ± 9.3 kg, height: 177.6 ± 6.3 cm. Participants were informed about the purposes, benefits, and risks before enrollment, and all agreed to participate and gave written informed consent. Approval was obtained (protocol number 94388718.8.0000.8093) from the Research Ethics Committee of the University of Brasília/Faculty of Ceilândia following the Helsinki Declaration of 1975. The present study is reported according to the CONSORT (Schulz et al., 2010).

### Participants

Participants were recruited through flyers distributed at the University and by verbal invitation. The inclusion criteria were: healthy males, aged 18–30 years, and physically active, but not engaged in systematic lower limb strengthening in the previous 6 months. The exclusion criteria were none responsiveness or discomfort with the NMES, considered as an MEIC lower than 40% of the MVC, and any musculoskeletal injury that could limit performance during the tests.

### Randomization and Allocation Concealment

**FIGURE 1:**
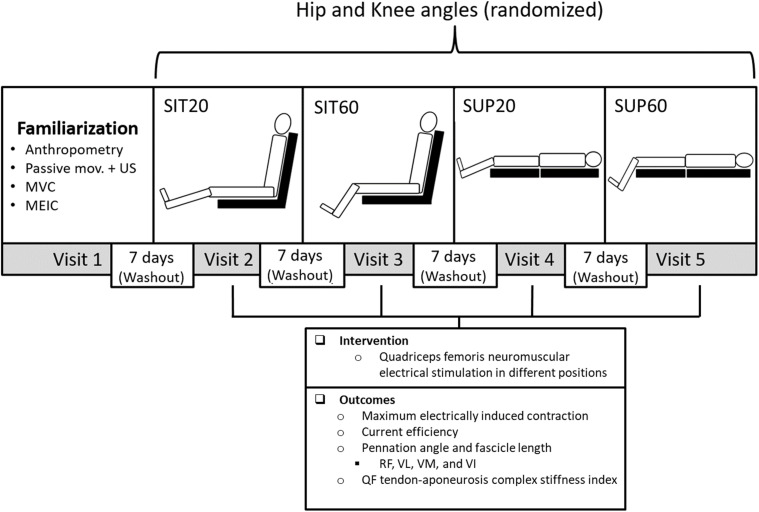
Experimental design: Participants took part in five sessions each at least 7 days apart: A familiarization and four experimental sessions to test four different combinations of hip and knee joint angles randomly during quadriceps femoris (QF) NMES. In the familiarization session, participants underwent anthropometric measurements, QF ultrasound imaging (US) during passive knee movement, MVC practice, and MEIC. In each experimental session, eight MEIC were required. The primary outcomes were the MEIC (absolute and normalized by the MVC) and the current efficiency (MEIC/current amplitude). The secondary outcome was the muscle architecture at rest and during NMES (pennation angle and fascicle length) of all QF constituents and the QF tendon-aponeurosis complex stiffness index. NMES, neuromuscular electrical stimulation; SUP60, supine with 60° of knee flexion; SIT60, seated with 60 of knee flexion; SUP20, supine with 20° of knee flexion; SIT20, seated with 20° of knee flexion, where supine was 0° of hip extension, seated was 85° of hip flexion, and full knee extension; RF, rectus femoris; VL, vastus lateralis; VM, vastus medialis; VI, vastus intermedius; MVC, maximum voluntary contraction; MEIC, maximum electrically induced contraction.

Four positions were randomly tested during NMES: lying supine with knee flexion of 60° (SUP60); seated with knee flexion of 60° (SIT60); lying supine with knee flexion of 20° (SUP20); and seated with knee flexion of 20° (SIT20), where supine was 0° of hip extension, seated was 85° of hip flexion, and full knee extension = 0° (Figure 1). One researcher prepared sealed, opaque, and numbered envelopes containing the order of testing. When each participant was enrolled in the study, the investigator opened the envelope with the numerical order. The positions were chosen considering that: (1) SIT60 is the position where the knee angle provides the ideal QF length for maximum torque production (Scott et al., 2019), and the hip angle provides a neutral length for the RF (Bampouras et al., 2017); (2) the QF is commonly stimulated with the knees fully extended (Fitzgerald et al., 2003). However, with the knees extended, it is not possible to measure the knee extensor torque properly on the isokinetic dynamometer (Babault et al., 2003). Therefore, we chose 20° knee flexion as an approximate position; (3) the hip angle affects the length of the RF and the myotendinous stiffness of the QF (Bampouras et al., 2017); therefore, extended and flexed conditions were used.

### Blinding

Participants were blinded to the study aim and hypotheses to avoid having their expectations affecting the performance. However, neither the researcher nor the volunteers could be blinded to the positions during the assessment.

### Interventions

This study involved five laboratory visits: a familiarization and four experimental sessions, 7 days of washout between each one, and conducted consistently between 09.00 AM and 4.00 PM. We instructed subjects not to ingest alcohol the previous 24 h or stimulants (e.g., caffeine, chocolate, and performance supplements) the previous 6 h before each visit. The familiarization consisted of: anthropometry (body mass and height); motor point localization on the VL and VM (Botter et al., 2011); and two MVC and two MEIC randomly performed in each position (in an isokinetic dynamometer; full detail on the subtopic *Torque assessment*), to verify if participants tolerated a current amplitude enough to generate a MEIC ≥ 40% of the MVC. The four positions were tested separately in each experimental session and consisted of eight MEIC during QF NMES. The timeline and content of the familiarization and experimental sessions are shown in Figure 1.

### Neuromuscular Electrical Stimulation

A neuromuscular electrical stimulator (Neurodyn 2.0, Ibramed, SP, Brazil) was connected to two isolated cables, each connected to a pair of self-adhesive electrodes of 25 cm^2^ applied over the motor points of the VL and VM (Botter et al., 2011). A biphasic pulsed current was used with frequency: 100 Hz, phase duration: 400 μs, ON time: 10 s (including a rise time of 3 s and a decay time of 3 s, and off time: 2 min). The specifications of the ON time was designed to mimic a ramp contraction and allow the QF muscle-tendon unit assessment with ultrasound imaging as recommended in voluntary contractions (Stafilidis and Tilp, 2015). The current amplitude was gradually increased while participants reported their discomfort using a 0–10 numeric scale after each NMES train, where 0 represented no discomfort and 10 represented the maximal perceived discomfort. Participants were informed that a report of 8 out of 10 of perceived discomfort should correspond to the maximum tolerated current amplitude they were willing to tolerate, according to a previous study (Scott et al., 2019). Moreover, trials at a given angle would end any time they wished to stop the testing. Eight MEIC in the right QF were elicited at a rate of 1 per minute. The mean of the first three MEIC was recorded and presented as absolute (N m) and relative (% of MVC) values. The mean current amplitude (mA) was used for the calculation of current efficiency as the ratio between absolute MEIC and current amplitude (Lieber and Kelly, 1991).

### Outcomes

The primary outcomes were the MEIC (absolute and normalized by MVC) and the current efficiency. Secondary outcomes were the muscle architecture at rest and during NMES (θ_*p*_ and *L*_*f*_) and the TAC stiffness of the four QF constituents (Figure 1).

### Torque Assessment

An isokinetic dynamometer (System 4; Biodex Medical Systems, Shirley, New York) were used to measure the knee extensor torque. The equipment axis was visually aligned with the knee flexion-extension axis. The knee and hip angles were determined using a goniometer, and the lever arm of the dynamometer transducer was firmly attached 2–3 cm above the lateral malleolus with a strap. A warm-up (six submaximal voluntary isometric contractions of 5 s) was performed prior to data collection. Before recording the evoked torque during NMES, participants completed two MVCs of the right knee extensors separated by a 2 min rest. During each MVC, participants were encouraged verbally to perform maximally and received visual feedback of the torque produced.

### Muscle Architecture

A linear probe (40 mm, 7.5 MHz, depth 6.0 cm, acquisition frame of 30 Hz) was connected to an ultrasound system (M-turbo, Sonosite, Washington, United States). With cine-loop ultrasound imaging, two recordings (from rest to MEIC plateau) were obtained of each QF constituent, and the best recording (better fascicle visualization) was used for analysis. For all ultrasound imaging outcomes, three measurements were performed and averaged. The probe was positioned longitudinally to the muscle fiber and perpendicular to the skin at 50% (RF), 60% (VL), 75% (VM), and 80% (VI), from proximal to the distal, of the distance between the medial aspect of the anterior superior iliac spine and the patella base, as adapted from previous reports (Blazevich et al., 2006; Massey et al., 2015). These regions were preferred because an isotropic muscle architecture and minimal fascicle curvature were expected (Blazevich et al., 2006). The RF and VI were visualized on the anterior aspect of the thigh, while the VL and VM were visualized, respectively, on the lateral and medial aspects. For the VI, although it could be seen on the same window of the RF or VL (Blazevich et al., 2006), VI visualization could be partially lost during contraction. Thus, it was recorded more distally. The probe was aligned so that the superficial and deep aponeuroses were parallel, and several fascicles could be delineated across the image (Baroni et al., 2013b; Geremia et al., 2019). Frames (at rest and MEIC plateau) of the recordings were saved as image files and analyzed in Image J software (v. 1.46; National Institutes of Health, Bethesda, United States. The best fascicle (i.e., the fascicle that could be clearly delineated from its insertion on the deep aponeurosis to the probe field-of-view limits) was used for Lf well as θ_*p*_ analysis (Geremia et al., 2019). The *L*_*f*_ was considered as the length of the fascicular path between superficial and deep aponeuroses. Thus, the remaining fascicle portion, from the field-of-view boundary to the superficial aponeurosis, was estimated by equation according to previous studies (Finni and Komi, 2002). The θ_*p*_ was calculated considering the angle between the deep aponeurosis and the fascicles. All measurements were conducted by the same investigator with extensive experience in ultrasonography.

### Tendon-Aponeurosis Complex Stiffness Index

The TAC displacement of each QF constituent was assessed using the same video recordings obtained for the extraction of muscle architecture variables. During data collection, a custom-made device held the probe, preventing it from moving. Moreover, the operator carefully maintained the device and probe to avoid any sliding further and maintain the best probe inclination concerning the skin surface along with contraction. If sliding occurred, it was corrected in relation to a hypoechoic shadow provided by an adhesive tape. Care was taken to limit compression on the skin surface. Moreover, ultrasonographic recordings were performed during passive motion of the knee from 60° to 0° in both seated and lying positions along with a digital goniometer (Miotec^®^, Porto Alegre/RS, Brazil) on the lateral aspect of the knee to correct displacement overestimation due to any knee joint angular rotation. Only the corrected values were used to calculate the stiffness of each constituent (Kubo et al., 2006).

**FIGURE 2:**
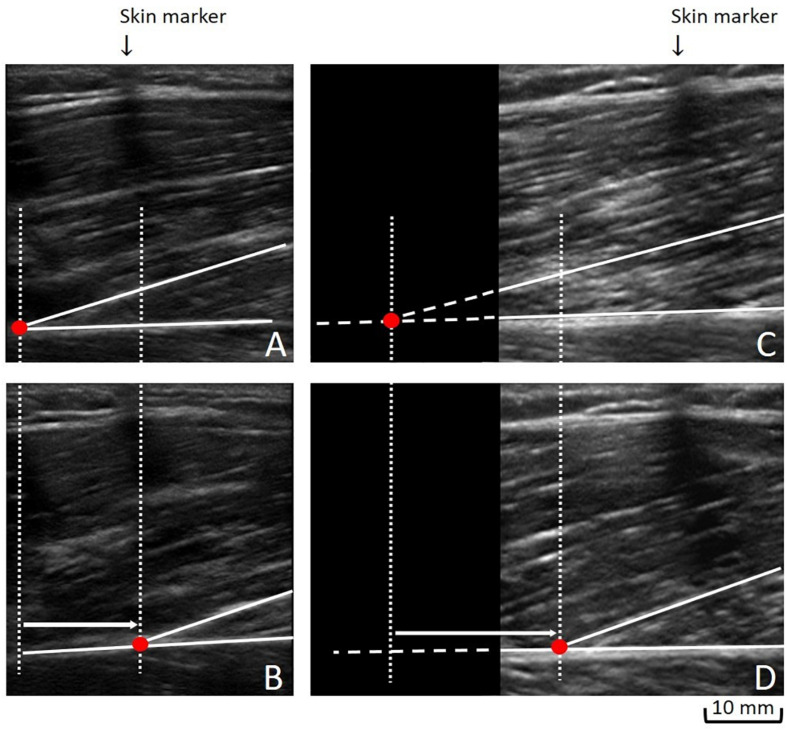
Measuring the tendon-aponeurosis complex displacement: To obtain the tendon-aponeurosis complex displacement during neuromuscular electrical stimulation of each quadriceps muscle constituent, two approaches could be used: (1) We followed a fascicle’ deep insertion from rest **(A)** to maximal evoked contraction plateau **(B)** along its visible path. (2) When a fascicle’ deep insertion could not be entirely followed from rest **(C)** to the maximal evoked contraction plateau **(D)**, a linear extrapolation was performed **(C)**.

We used the Tracker 4.87 software to manually track the cross point between the fascicle and the deep aponeurosis, and its displacement in millimeters from rest (Figures 2A,B), to contraction (Figures 2C,D). Due to the possibility of its deep insertion starting outside the probe’s field-of-view, we commonly made linear extrapolation, which is a standardized method (Ando et al., 2014; Massey et al., 2015). Stiffness calculation uses force (N) as unit. Thus, to calculate the QF evoked force, the knee extensor torque values during MEIC were multiplied by the internal moment arm, which was standardized for all participants according to the knee angle (60°: 0.056 m; 20°: 0.0475 m) (Krevolin et al., 2004). To obtain a QF TAC stiffness index, we used the delta from 40 to 100% of the MEIC and the delta displacement of each QF constituent at the respective levels of force (40 and 100%). This procedure was conducted assuming that no tendon slack would be present at this force level. Once we were unable to estimate the contribution to the evoked force of each constituent, the evoked force was divided by the averaged displacement values as follows: QF TAC stiffness index = (100% of evoked force – 40% of evoked force)/[TAC displacement of RF + VL + VM + VI]/4).

Furthermore, to synchronize the MEIC and ultrasonographic recordings, we used a data acquisition device, New Miotool (Miotec Biomedical Equipment Ltd., POA, Brazil^®^) collected with a sampling rate of 2,000 Hz per channel, A/D converter of 14 bits, common rejection mode of 110 db (at 60 Hz). For this, the data acquisition device was connected to the computerized dynamometer, and a high-definition camera was positioned to capture the ultrasound display. When the assessor started recording cine-loop ultrasound images prior to the MECs, a visual indicator appeared on the ultrasound screen, which enabled the synchronization of all data on a torque-time recording generated by the device (Bojsen-Moller et al., 2003).

### Statistical Analysis and Sample Size

All outcomes are reported as mean and 95% confidence interval (95% CI). Repeated measures one-way ANOVA with a within-subject of “*position*” (SUP60, SIT60, SUP20, and SIT20) was applied to verify differences between positions for MVC, maximal evoked torque, current efficiency, current amplitude, and TAC stiffness index. A two-way ANOVA [“*position*” (4 levels: SUP60, SIT60, SUP20, and SIT20) by “*condition*” (2 levels: rest and MEIC)] was applied to verify changes in θ_*p*_ and *L*_*f*_. When a significant difference was detected, a Tukey *post hoc* test was applied to identify the differences. Effect sizes (partial eta squared −η_ρ_^2^) and statistical power were provided. For reliability of consecutive MEIC, a mean of multiple measurements, absolute-agreement, 2-way mixed-effects model was used for intra-class correlation (ICC) (the torque of all eight contractions performed in each position). Moreover, a single-measurement, absolute-agreement, 2-way mixed-effects model was used for the ICC of *L*_*f*_, θ_*p*_, and TAC displacement (two repeated analyses, seven to 14 between-days, of 25 recordings for each QF constituent). Reliability was classified as: poor (<0.5), moderate (0.5–0.75), good (>0.75–0.9), and excellent (>0.9) (Koo and Li, 2016). Coefficient of Variation (CV) was provided for each reliability analysis (mean ± standard deviation). The sample size was determined using G^∗^Power (version 3.1.3; University of Trier, Trier, Germany) with the level of significance set at *p* = 0.05 and power (1−β) = 0.80 to detect a large effect (η_ρ_^2^ > 0.14). According to a previous study (Scott et al., 2019), we estimated a difference between means and standard deviation of 123.7 ± 35.7 Nm and 222.6 ± 67.1 (evoked torque) in the knee at 30° and 60°, respectively. The significance threshold was set at α < 0.05. All statistical analyses were performed using Statistica 23.0 (STATSOFT Inc., Tulsa, Oklahoma, United States).

## Results

**FIGURE 3:**
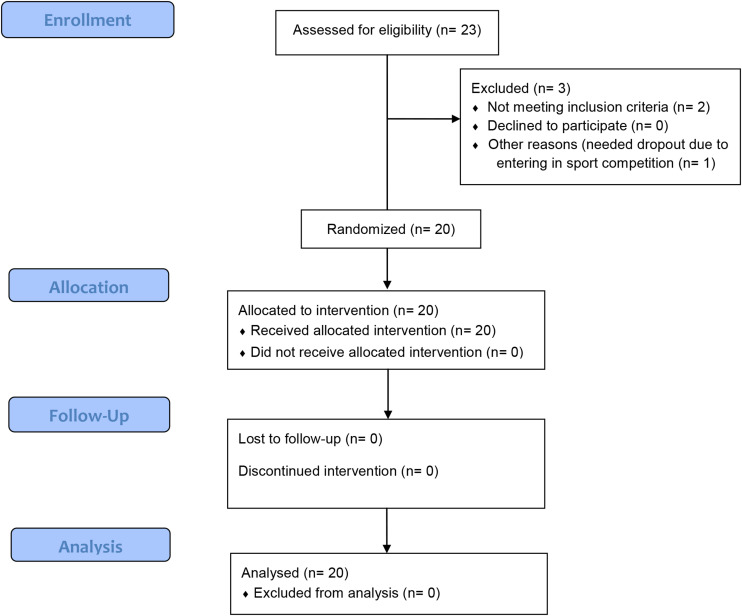
Flowchart of the randomized single-blind study.

Twenty-three men were evaluated for eligibility criteria, and twenty men were included for randomization and completed the study. No cases of skin burn, or injury caused by NMES, occurred. Figure 3 shows a detailed flowchart of participant selection, allocation, follow-up, and analysis.

### Reliability of Measurements

ICC was excellent for evoked torque at SUP60 (0.99; CV: 0.07 ± 0.04%), SIT60 (0.99; CV: 0.08 ± 0.05%), SUP20 (0.99; CV: 0.05 ± 0.04%), and SIT20 (0.99; CV: 0.05 ± 0.04%). We obtained good reliability for the θ_*p*_ of RF (0.75; CV: 10.03 ± 7.34%), VL (0.78; CV: 6.77 ± 6.12%), VM (0.82; CV: 8.12 ± 7.33%), and VI (0.77; CV: 8.47 ± 6.85%), and good reliability for the *L*_*f*_ of RF (0.81; CV: 12.49 ± 8.40%), VL (0.80; CV: 7.33 ± 7.59%), VM (0.77; CV: 5.41 ± 5.32%), and VI (0.79; CV: 7.51 ± 8.15%). For the TAC displacement, the ICC was good to excellent for RF (0.98; CV: 1.83 ± 2.07%), VL (0.95; CV: 4.71 ± 6.90%), VM (0.95; CV: 2.67 ± 1.12%), and VI (0.86; CV: 3.05 ± 3.06%).

### MVC, MEIC (Absolute and Normalized), Current Amplitude, and Current Efficiency

Table 1 shows the mean (95% CI), significance, power, and η_ρ_^2^ for MVC, MEIC (absolute and normalized), current amplitude, and current efficiency. There was a significant main effect for MVC (*p* < 0.001), MEIC (*p* < 0.001), and current efficiency (*p* < 0.001). SUP60 and SIT60 showed greater MVC (*p* < 0.001), absolute evoked torque (*p* < 0.001), and current efficiency (*p* < 0.01) than SUP20 and SIT20. For these variables, no differences were found when we compared positions with the same knee angle (SIT60 vs. SUP60: *p* = 0.71, 0.51, and0.15, respectively; SIT20 vs. SUP20: *p* = 0.99,0.98, and0.49, respectively). For normalized MEIC, there was no main effect among positions (*p* = 0.14). Current amplitude presented a significant main effect among positions (*p* < 0.001). A lower current amplitude was found at SUP20 compared to SIT60 (*p* < 0.001), SIT20 (*p* < 0.03), and SUP60 (*p* < 0.001), and SIT60 was greater than SIT20 (*P* < 0.02). SUP60 did not differ from SIT60 and SIT20 (*P* = 0.45). All participants reported 8 out 10 of perceived discomfort that was considered the maximum tolerated current amplitude.

**TABLE 1:** Maximal voluntary contraction (MVC), absolute, and normalized maximum electrically induced contraction (MEIC), current amplitude, and current efficiency at different hip and knee angles.

	SUP60	SIT60	SUP20	SIT20	One-way ANOVA (*Position* effect)		
					*P*-value	Power	Partial ηρ^2^
MVC (N m)	212.34 (196.28–236.63)	201.45 (184.21–228.86)	95.43 (88.78–105.14)^*a,b*^	93.13 (84.55–107.21)^*a,b*^	<.001	1.0	0.84
MEIC (N m)	162.19 (143.27–201.78)	142.59 (127.73–183.42)	67.24 (60.50–82.27)^*a,b*^	62.01 (54.84–79.19)^*a,b*^	<0.001	1.0	0.68
Normalized evoked torque (%)	76.38 (68.92–92.70)	70.78 (64.05–85.33)	70.46 (64.39–80.66)	66.58 (60.40–78.12)	0.20	0.39	0.07
Current amplitude (mA)	78.68 (71.16–91.76)	84.04 (75.45–99.05)	61.83 (55.97–72.35)^*a,b*^	71.09 (62.37–88–92)^*b,c*^	<0.001	0.99	0.40
Current efficiency (N m/mA)	2.06 (1.85–2.46)	1.69 (1.51–2.11)	1.08 (0.95–1.34)^*a,b*^	0.87 (0.76–1.07)^*a,b*^	<.0010.001	1.0	0.56

**Values are reported as mean and 95% CI. Abbreviations: supine with knee flexion of 60*° *(SUP60); seated with knee flexion of 60*° *(SIT60); supine with knee flexion of 20*° *(SUP20); and seated with knee flexion of 20*° *(SIT20), where supine was 0*° *of hip extension, seated was 85*° *of hip flexion, and full knee extension = 0*°. *^*a*^P < 0.05 vs. SUP60- ^*b*^P < 0.05 vs. SIT60- ^*c*^P < 0.05 vs. SUP20.**

### Muscle Architecture

Supplementary Table 1 shows the values of muscle thickness, θ_*p*,_ and *L*_*f*_ of the QF constituents at rest and during MEIC and the ANOVA results (except for muscle thickness). Figure 3 presents the values of θ_*p*_ and *L*_*f*_ at rest and (as well as the main effect of position) during evoked contraction for the QF constituents, along with the *post hoc* significance.

**FIGURE 4:**
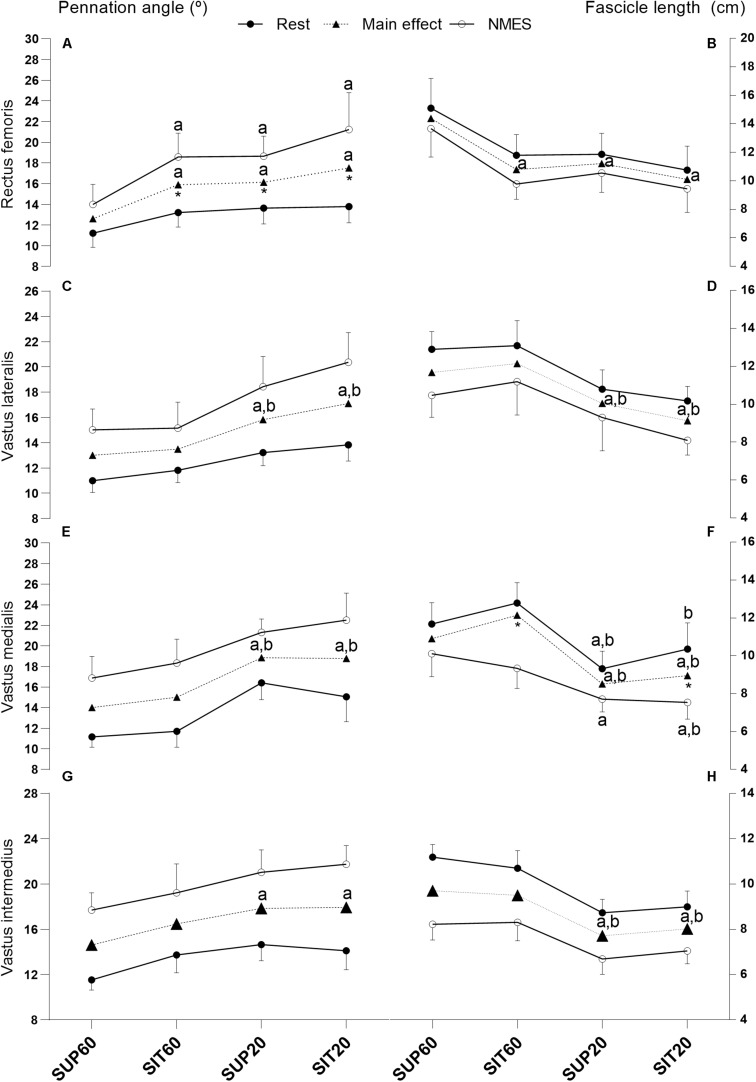
Muscle architecture changes of the quadriceps femoris constituents in different muscle lengths according to hip and knee angles at rest and during NMES: Pennation angle (left *y* axis) and fascicle length (right *y* axis) of all constituents of the quadriceps femoris individually and grouped at rest, during NMES (continuous lines), and main effect of position (dotted lines). Data are presented as mean and 95% CI. **(A,B)** *Rectus femoris*- **(C,D)** *Vastus lateralis*- **(E,F)** *Vastus medialis-* **(G,H)** *Vastus intermedius*. Legend: NMES, neuromuscular electrical stimulation; SUP60, supine with 60° of knee flexion; SIT60, seated with 60° of knee flexion; SUP20, supine with 20° of knee flexion; SIT20, seated with 20° of knee flexion, where supine was 0° of hip extension, seated was 85° of hip flexion, and full knee extension. Statistically significant differences: ^*a*^different from SUP60; ^*b*^different from SIT60. *Indicate significant difference (*p* ≤ 0.05) between rest and MEIC when there was a position by time effect. The significance threshold was set at α < 0.05.

For RF (Figures 4A,B), there was an interaction effect between *position* and *intensity* for θ_*p*_ (*p* = 0.017). In comparison to rest, NMES promoted higher θ_*p*_ in SIT60, SUP20, and SIT20 (*p* < 0.001). During MEIC, SUP60 presented lower θ_*p*_ compared to SIT60, SUP20, and SIT20 (*p* < 0.001). There was no interaction of factors for *L*_*f*_ (*p* = 0.90), but position effect was significant (*p* < 0.001). SUP60 presented greater *L*_*f*_ (*p* < 0.001–0.002) than other positions.

Considering VL (Figures 4C,D), there were no significant interaction effect for θ_*p*_ (*p* = 0.095) or *L*_*f*_ (*p* = 0.79). Position factor was significant for both θ_*p*_ (*p* < 0.001) and *L*_*f*_ (*p* < 0.001). The *post hoc* analysis showed lower θ_*p*_ (*p* < 0.001–0.011) and greater *L*_*f*_ (*p* < 0.001–0.031) for SUP60 and SIT60 compared to SUP20 and SIT20.

VM (Figures 4E,F) demonstrated a significant interaction effect for *L*_*f*_ (*p* = 0.044). The *post hoc* showed that SIT60 (*p* < 0.001) and SIT20 (*p* < 0.001) were different from rest to contraction, but not SUP60 (*p* = 0.10) and SUP20 (*p* = 0.083). Moreover, comparing positions, *L*_*f*_ was significantly greater for most analyses of SUP60 and SIT60 compared to SUP20 and SIT20 (*p* < 0.001 –0.03), except SUP60 vs. SIT20 at rest (*p* = 0.25) and SIT60 vs. SUP20 during NMES (*p* = 0.077). There was no interaction for θ_*p*_ (*p* = 0.097), but there was a significant main effect of *position* (*p* < 0.001). SUP60 and SIT60 showed lower θ_*p*_ (*p* < 0.001) compared to SUP20 and SIT20.

For VI (Figures 4G,H), there was no significant effect of interaction for θ_*p*_ (*p* < 0.25) or *L*_*f*_ (*p* = 0.15), but position factor was significant for both θ_*p*_ (*p* < 00.1) and *L*_*f*_ (*p* < 0.001). *L*_*f*_ was greater at SUP60 and SIT60 than SUP20 and SIT20 (*p* < 0.001). However, for θ_*p*_, SUP60 was greater than SUP20 (*p* < 0.001) and SIT20 (*p* < 0.001), but SIT60 was not (*p* = 0.25 and 0.30, respectively).

**FIGURE 5:**
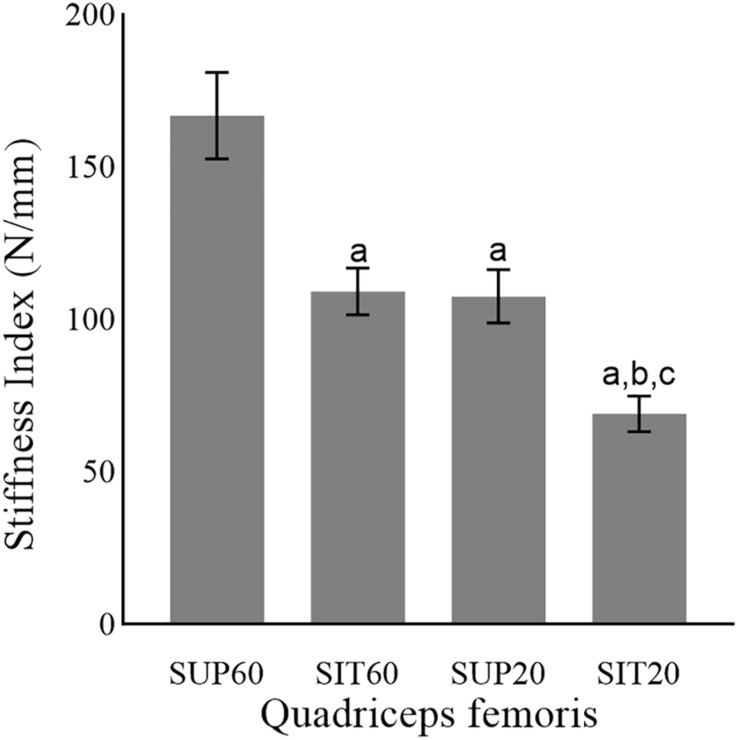
The tendon-aponeurosis complex stiffness index of the quadriceps femoris in different muscle lengths according to hip and knee angles during neuromuscular electrical stimulation. Abbreviations: SUP60, lying with 60° of knee flexion; SIT60, seated with 60° of knee flexion; SUP20, lying with 20° of knee flexion; SIT20, seated with 20° of knee flexion. Statistically significant differences: ^*a*^*P* < 0.05 vs. SUP60; ^*b*^*P* < 0.05 vs. SIT60. ^*c*^*P* < 0.05 vs. SUP20. Data are presented as mean and 95% CI.

There was a significant main effect of position (*p* < 0.001 ηρ^2^: 0.50, power: 0.99) for the TAC stiffness index (Figure 5). In SUP60 the stiffness index was greater than for all positions (*p* < 0.001). SIT60 and SUP20 were not different (*p* = 0.99). For SIT20, the stiffness index was also lower than in SUP20 (*p* = 0.02) and SIT60 (*p* = 0.01).

## Discussion

The main findings of this study were: (1) MEIC and current efficiency were greater at 60° than 20° of knee flexion. At these knee angles, changing the hip angle (neutral or flexed at 85°) did not affect torque output; (2) the QF constituents showed lower θ_*p*_ and greater *L*_*f*_ at 60° of knee flexion, but for the RF this only occurred when the hip was extended. Moreover, during MEIC, *L*_*f*_ reduction of VM was lower in supine; (3) The TAC stiffness index was greater in SUP60. These new findings may help exercise physiologists and clinicians to develop effective strategies when applying NMES by choosing a knee angle around 60° when the goal is to induce high MEIC of knee extension. Furthermore, the QF elongation at the hip may be chosen to provide greater mechanical stress to the muscle-tendon unit.

We demonstrated that NMES produces greater MEIC of knee extension and current efficiency applied with the knee at 60° compared to 20° irrespective of hip angle. Our results are in agreement with previous reports that found higher MEIC at 60° of knee flexion compared to more extended positions (15° and 30°) (Bremner et al., 2015; Scott et al., 2019). In addition, current efficiency was greater at SUP60 and SIT60, demonstrating that NMES generated higher torques with lower current amplitude (Lieber and Kelly, 1991; Medeiros et al., 2017). Our protocol required the maximum tolerated current amplitude. However, at a chosen percentage of the MVC, a cautiously chosen joint angle could allow the targeted torque with lower current amplitude and less sensory discomfort, which is a common limitation of NMES (Thomas and Stevens-Lapsley, 2012; Herzig et al., 2015). Collectively, it is possible to suggest that NMES should be applied at 60° of knee flexion unless contraindicated by disease or not tolerated.

The QF constituents mostly operated with lower θ_*p*_ and greater *L*f when the knee was flexed at 60° compared to 20°. It is possible that the positions SUP60 and SIT60 placed the QF at a better physiologic architectural configuration for torque generation, i.e., allowed improved transmission of the muscle force to the tendon and ideal sarcomere/fiber length (Gordon et al., 1966; Fukunaga et al., 1997; Lieber and Friden, 2000). In contrast, SUP20 and SIT20 caused an increased θ_*p*_ and shorter *L*f, which, respectively, attenuates the force transmission to bones (Massey et al., 2015) and reduces force production according to the force-length relationship (Herzog et al., 1990). Interestingly, in regards to differences from rest to MEIC, for most comparisons (except θ_*p*_ of RF and *L*_*f*_ of VM; Figures 3A,F), they were similar across positions, despite different torque values, indicating that the amount of muscle shortening is also dependent on the slack in the muscle-tendon unit (Raiteri, 2018).

We demonstrated a clear effect of a supine position on the RF (Figures 3A,B), as expected for the biarticular constituent of QF. The θ_*p*_ was lower and *L*_*f*_ was higher at SUP60 than in all other positions. The similarity between SIT60 and SUP20 probably occurred because each position shortened the RF in one joint and lengthened it at another. Furthermore, the similarity between SUP20 and SIT20 may indicate that no significant reduction occurs in fiber length beyond a certain angle within the range of motion despite increased slack of the muscle-tendon unit (Fukunaga et al., 1997). This is supported by Herzog et al. (1990), who predicted that the RF force generation capacity ceases before full knee extension is reached (when the hip is flexed), due to active insufficiency.

VL and VM were affected by changing the knee angle, i.e., lower θ_*p*_ and greater *L*_*f*_ when the knee was at 60° compared to 20°, as expected (Figures 3C–F). Unexpectedly, from rest to MEIC, Lf reduction of the VM was lower in the supine positions. This occurred from a start point (resting state) where the *L*_*f*_ was non-significantly reduced compared to a seated position. A previous study reported that the VM insertion expands over the VI aponeurosis and on the RF medial edge (Grob et al., 2018). Besides, interaction between synergistic muscles is known as the existence of interaction caused by inter-and extramuscular connective tissues (Maas and Sandercock, 2010). Considering these anatomical descriptions, it is possible to speculate that lengthening the RF at the hip would, at the same time, promote a shortening effect on the VM. Once this specific VM shortening occurs while the RF is elongating the tendinous tissues, then the VM fascicles are unable to shorten expressively in supine. Future studies may consider the effect of hip angle on the VM loading. For the VI, only SUP60 demonstrated lower θ_*p*_ than the positions with the knee at 20° (Figure 3G). Since the VI is surrounded by superficial muscles (RF, VL, and VM), it may be compressed due to space constraints (Lieber and Friden, 2000). Therefore, when the RF is stretched at SUP60, it probably compresses the VI and reduces its θ_*p*_.

The QF had greater TAC stiffness index in SUP60 (more elongated position) than all other positions (Figure 4), indicating an increased passive tension that limited tendinous elongation during contraction (Fukutani et al., 2017). The increased tension of the TAC in stretched conditions is known to allow stronger contractions with less effort due to better force transmission (Abellaneda et al., 2009; Raiteri, 2018). However, we did not assess the electromechanical delay (Begovic et al., 2014), which could elucidate this effect in the present study. Interestingly, current efficiency is progressive higher from the more shortened (SK20), to the most elongated (SUP60) position. Thus, a plausible reason may be the best use of the current amplitude by a stiffer muscle-tendon unit.

Earlier studies (Maffiuletti and Lepers, 2003; Bampouras et al., 2017) found greater knee extensor MEIC in supine than seated with the knee flexed at 90°. Here, we only found a non-significant increase in the MEIC and current efficiency at SUP60 compared to SIT60. Others have found higher triceps sural torque at a longer muscle length compared to a neutral position (Fukutani et al., 2017). However, for the QF, a more stretched position at 90° of knee flexion decreased voluntary and evoked torque compared to 60° (Gerrits et al., 2005; Scott et al., 2019). These discrepancies indicate a limit between improving force transmission while avoiding mismatches between contractile filaments of muscle fibers for different muscle-tendon units (Herzog et al., 1990). Thus, a fundamental knowledge of muscle-tendon unit physiology is crucial to the understanding, rationales, and appropriate NMES application in the different hip and knee joint angles.

Stiffer tendons promote less muscle shortening at the same absolute force (Massey et al., 2015). Accordingly, we found that the increase in θ_*p*_ of the RF was not significant at SUP60 (Figure 3A), and neither was the reduction in *L*_*f*_ of the VM at SUP60 and SUP20 (Figure 3F), corresponding to increased stiffness in these positions (Figure 4). Indeed, in SUP20, there was a remarkable increase in the stiffness index once it was greater than SIT20 and not different from SIT60. On the other hand, greater fascicle shortening is necessary to eliminate the TAC slack in more shortened positions (Fukunaga et al., 1997), explaining why SUP20 and SIT20 changes from rest to contraction on θp and Lf were mostly similar to that observed at SUP60 and SIT60 despite torque dissimilarities. Suydam et al. (2015) showed that a surgically lengthened Achilles tendon reduces the triceps surae muscle ability to generate adequate output, requiring more muscle shortening without efficient load transmission. Similarly, it is possible that a muscle in a shortened position needs to contract more without significant load, limiting the mechanical stress necessary to drive strengthening and hypertrophy. Future integrative physiological studies will be required to provide insights into the mechanisms by which such adaptations occur.

Some limitations should be addressed in the present study. One limitation is related to the behavior of the NMES-induced contractions assessed by ultrasound. The visualization of NMES contraction is challenging due to muscle deformation and reduced control of contraction velocity, which we attempted to attenuate by an automatic 3 s rise on current amplitude, mimicking a ramp contraction (Stafilidis and Tilp, 2015). Despite this limitation, we showed moderate to excellent ICC reliability and low CV for the ultrasound imaging outcomes. Another limitation, we could not determine this relative contribution of QF constituents to knee extensor torque during NMES. Future studies may address this question. Previous reports determined the relative contribution during voluntary contraction (Zhang et al., 2003). However, it was not possible to extrapolate these results to evoked contractions in different muscle lengths according to joint angle. Finally, our results are also limited to our population and for a single session of NMES. Further studies are necessary to elucidate the influence of the angle-torque association between muscle architecture and tendon-aponeurosis behavior during an NMES training session and in clinical populations.

## Conclusion

NMES generate greater MEIC and current efficiency at 60°, compared to 20° of knee flexion. For these knee angles, lengthening QF at the hip did not promote significant change. Although a greater *L*_*f*_ and lower θ_*p*_ were predominant in SUP60 and SIT60, each QF constituent demonstrated a unique behavior in different muscle lengths. QF TAC stiffness index increased in more elongated positions, which probably contributed to enhanced force transmission and slightly higher torque in SUP60.

## Data Availability Statement

The original contributions presented in the study are included in the article/Supplementary Material, further inquiries can be directed to the corresponding author/s.

## Ethics Statement

Approval for this study involving human participants was obtained (protocol number 94388718.8.0000.8093) from the Research Ethics Committee of the University of Brasília/Faculty of Ceilândia. The participants provided their written informed consent to participate in this study.

## Author Contributions

JC, RM, KS, MB, NB, and JD conceived and designed research. JC performed the experiments and drafted the manuscript. JC, NB, and JD analyzed the data. JC, RM, JG, IS, BB, KS, MB, NB, and JD interpreted the results of experiments, edited and revised the manuscript, and approved the final version of the manuscript. JC and IS prepared the Figures. All authors contributed to the article and approved the submitted version.

## Conflict of Interest

The authors declare that the research was conducted in the absence of any commercial or financial relationships that could be construed as a potential conflict of interest.

## Abbreviations

MEIC: maximum electrically induced contraction
MVC: maximum voluntary contraction
TAC: tendon aponeurosis complex
θ_*p*_: pennation angle
*L*_*f*_: fascicle length
NMES: neuromuscular electrical stimulation
RF: rectus femoris
VL: vastus lateralis
VM: vastus medialis
VI: vastus intermedius
QF: quadriceps femoris
SIT60: seated with 60° of knee flexion
SIT20: seated with 20° of knee flexion
SUP60: supine with 60° of knee flexion
SUP20: supine with 20° of knee flexion
ANOVA: analysis of variance
ICC: intra-class correlation
CI: confidence interval
CV: coefficient of variation.
