# Supplementary material for: The Effect of Quadriceps Muscle Length on Maximum Neuromuscular Electrical Stimulation Evoked Contraction, Muscle Architecture, and Tendon-Aponeurosis Stiffness
Source: Front Physiol. 2021 Mar 29;12:633589. doi: 10.3389/fphys.2021.633589 (PMC8040804; doi:10.3389/fphys.2021.633589)
Supplement: Supplementary file 1 [file Table_1.docx]

| **Supplementary Table 1 -** Muscle thickness, fascicle length, and pennation angle of rectus femoris, vastus lateralis, vastus medialis, and vastus intermedius at rest and during neuromuscular electrical stimulation in four combinations of hip and knee joint angles | | | | | | | | | | | | |
| --- | --- | --- | --- | --- | --- | --- | --- | --- | --- | --- | --- | --- |
|  | **SUP60** | | **SIT60** | | **SUP20** | | **SIT20** | |  |  |  |  |
|  | Rest | NMES | Rest | NMES | Rest | NMES | Rest | NMES | Two-way ANOVA  (position by condition) | P | Power | Partial ηρ2 |
| **Rectus femoris** | | | | | | | | |  |  |  |  |
| MT | 2.61 (2.45 - 2.82) | 2.96 (2.77 - 3.23) | 2.43 (2.29- 2.63) | 2.87 (2.69- 3.14) | 2.44 (2.27- 2.67) | 3.14 (2.89 - 3.49) | 2.43 (2.26 - 2.66) | 3.04 (2.81 - 3.38) |  |  |  |  |
|  |  | |  | |  | |  | |  |  |  |  |
| θ_p_ | 10.87 (9.85 - 12.58) | 13.45 (12.05 - 15.94) | 12.90 (11.81- 14.60) | 18.0 (16.27- 20.88) | 13.27 (12.12 - 15.15) | 18.23 (16.69 - 20.59) | 13.41 (12.22 - 15.33) | 20.0 (17.66 - 24.79) | Interaction | 0.017 | 0.77 | 0.16 |
|  |  |  |  |  |  |  |  |  | Main effect (*position*) | < 0.001 | 0.98 | 0.28 |
| *L*_f_ | 14.40 (13.00 - 17.19) | 13.01 (11.67 - 15.66) | 11.41 (10.35- 13.24) | 9.53 (8.71- 10.84) | 11.47 (10.39 - 13.34) | 10.11 (9.20 - 11.88) | 10.26 (9.06 - 12.44) | 8.65 (7.79 - 11.09) | Interaction | 0.908 | 0.08 | 0 |
|  |  |  |  |  |  |  |  |  | Main effect (*position*) | <0.001 | 0.99 | 0.33 |
| **Vastus lateralis** | | | | | | | | |  |  |  |  |
| MT | 2.41 (2.30 - 2.55) | 2.63 (2.43 - 2.89) | 2.49 (2.36- 2.64) | 2.70 (2.55- 2.90) | 2.19 (2.06 - 2.37) | 2.65 (2.47 - 2.92) | 2.30 (2.19 - 2.44) | 2.65 (2.52 - 2.82) |  |  |  |  |
|  |  |  |  |  |  |  |  |  |  |  |  |  |
| θ_p_ | 10.83 (10.06 - 11.91) | 14.61 (13.35 - 16.67) | 11.64 (10.85- 12.77) | 14.53 (13.09- 17.21) | 13.04 (12.18- 14.26) | 17.70 (16.02- 20.83) | 13.58 (12.55 - 15.11) | 19.79 (18.01 - 22.72) | Interaction | 0.095 | 0.53 | 0.10 |
|  |  |  |  |  |  |  |  |  | Main effect (*position*) | <0.001 | 0.99 | 0.42 |
| *L*_f_ | 12.73 (11.94 - 13.83) | 10.19 (9.31- 11.62) | 12.81 (11.75- 14.41) | 10.64 (9.43- 12.94) | 10.60 (9.77- 11.81) | 8.75 (7.54 - 11.04) | 10.03 (9.38 - 10.95) | 7.92 (7.32 - 8.86) | Interaction | 0.799 | 0.11 | 0.01 |
|  |  |  |  |  |  |  |  |  | Main effect (*position*) | <0.001 | 0.99 | 0.38 |
| **Vastus medialis** | | | | | | | | |  |  |  |  |
| MT | 2.25 (2.11- 2.44) | 2.85 (2.63 - 3.18) | 2.46 (2.29- 2.70) | 2.79 (2.58- 3.10) | 2.34 (2.19 - 2.56) | 2.90 (2.72 - 3.15) | 2.46 (2.31 - 2.67) | 2.85 (2.69 - 3.05) |  |  |  |  |
|  |  |  |  |  |  |  |  |  |  |  |  |  |
| θ_p_ | 10.95 (10.15 - 12.14) | 16.31 (14.76 - 18.98) | 11.26 (10.16- 13.23) | 17.6 (15.99- 20.65) | 16.07 (14.78 - 18.03) | 21.12 (19.98 - 22.61) | 14.11 (12.62 - 17.47) | 21.78 (19.85 - 25.13) | Interaction | 0.097 | 0.53 | 0.10 |
|  |  |  |  |  |  |  |  |  | Main effect (*position*) | <0.001 | 0.99 | 0.43 |
| *L*_f_ | 11.45 (10.54 - 12.80) | 9.81 (8.90- 11.32) | 12.58 (11.71- 13.86) | 9.08 (8.28- 10.39) | 9.13 (8.39 - 10.24) | 7.58 (7.04 - 8.37) | 9.91 (8.96- 11.74) | 7.30 (6.66- 8.41) | Interaction | 0.044 | 0.65 | 0.13 |
|  |  |  |  |  |  |  |  |  | Main effect (*position*) | <0.001 | 0.99 | 0.40 |
| **Vastus intermedius** | | | | | | | | |  |  |  |  |
| MT | 2.29 (2.16 - 2.47) | 2.34 (2.22 - 2.49) | 2.23 (2.09- 2.41) | 2.44 (2.31- 2.62) | 2.06 (1.91 - 2.28) | 2.23 (2.08 - 2.44) | 1.81 (1.68 - 2.0) | 2.35 (2.24 - 2.49) |  |  |  |  |
|  |  |  |  |  |  |  |  |  |  |  |  |  |
| θ_p_ | 11.38 (10.63 - 12.43) | 17.43 (16.17 - 19.22) | 13.38 (12.15- 15.30) | 18.5 (16.67- 21.77) | 14.34 (13.23 - 16.05) | 20.63 (19.05 - 23.02) | 13.71 (12.42 - 15.77) | 21.48 (20.10 - 23.39) | Interaction | 0.253 | 0.35 | 0.06 |
|  |  |  |  |  |  |  |  |  | Main effect (*position*) | <0.001 | 0.98 | 029 |
| *L*_f_ | 11.12 (10.61 - 11.74) | 8.10 (7.53 - 8.90) | 10.56 (9.91- 11.47) | 8.11 (7.49- 9.11) | 8.64 (8.13 - 9.32) | 6.54 (6.01- 7.36) | 8.87 (8.28 - 9.69) | 6.94 (6.48- 7.59) | Interaction | 0.175 | 0.42 | 0.08 |
|  |  |  |  |  |  |  |  |  | Main effect (*position*) | <0.001 | 1.0 | 0.54 |
| Values are expressed as mean (95% CI). Statistical analyses showed only for θ_p_  and *L*_f_. Legend: SIT20: seated with knee at 20º- SIT60: seated with knee at 60º- SUP20: supine with knee at 20º- SUP60: supine with knee at 60º- MT: muscle thickness- θ_p_: pennation angle-  *L*_f_ : fascicle length. | | | | | | | | | | | | |
